# 2-{[3-Methyl-4-(2,2,2-trifluoro­eth­oxy)pyridin-2-yl]methyl­sulfan­yl}-1*H*-benzimidazole monohydrate: a monoclinic polymorph

**DOI:** 10.1107/S1600536812022131

**Published:** 2012-06-13

**Authors:** Yu-Feng Chen, Jin-Yao Chen, Ming-Huang Hong, Jie Lu, Guo-Bin Ren

**Affiliations:** a School of Chemical Engineering and Energy, Zhengzhou University, Zhengzhou 450001, People’s Republic of China; b Pharmaceutical Crystal Engineering Research Group, Shanghai Institute of Pharmaceutical Industry, 1320 Beijing Road (West), Shanghai 200040, People’s Republic of China; c National Engineering Laboratory for Cereal Fermentation Technology, School of Chemical & Material Engineering, Jiangnan University, Wuxi 214122, People’s Republic of China

## Abstract

The title compound, C_16_H_14_F_3_N_3_OS·H_2_O, which had been previously characterized in the space group *P*-1 [Ren *et al.* (2011 ▶). *Acta Cryst*. E**67**, o270], has now been crystallized from 1-propanol in the monoclinic form in the space group *P*2_1_/*c*. While the triclinic form is a *Z*′ = 2 crystal, the new monoclinic polymorph includes one main mol­ecule and one water lattice mol­ecule in the asymmetric unit. In the crystal, the water mol­ecule is sandwiched between neighboring main mol­ecules and behaves as both donor and acceptor in O—H⋯N and N—H⋯O hydrogen bonds with the imidazole N atoms. This pattern of chains parallel to [100] further inter­acts *via* O—H⋯N(pyridine) contacts.

## Related literature

For the role of the title compound in the synthesis of the anti-ulcer drug lansoprazole {systematic name: (*RS*)-2-([3-methyl-4-(2,2,2-trifluoro­eth­oxy)pyridin-2-yl]methyl­sulfin­yl)-1*H*-benzo[*d*]imidazole}, see: Del Rio *et al.* (2007 ▶); Reddy *et al.* (2008 ▶); Iwahi *et al.* (1991 ▶). For related structures, see: Swamy & Ravikumar (2007 ▶); Hakim Al-arique *et al.* (2010 ▶). For the triclinic polymorph of the title hydrate, see: Ren *et al.* (2011 ▶) and for the structure of the propan-2-ol solvo-polymorph, see: Ma *et al.* (2012 ▶)
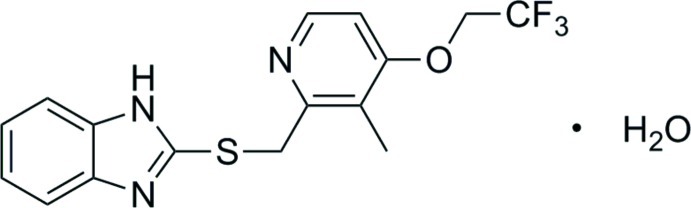


## Experimental

### 

#### Crystal data

C_16_H_14_F_3_N_3_OS·H_2_O
*M* *_r_* = 371.39
Monoclinic, 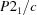

*a* = 7.3886 (15) Å
*b* = 25.497 (5) Å
*c* = 8.8579 (18) Å
β = 93.64 (3)°
*V* = 1665.4 (6) Å^3^
*Z* = 4
Cu *K*α radiation
μ = 2.17 mm^−1^
*T* = 296 K
0.27 × 0.16 × 0.15 mm

#### Data collection

Bruker SMART APEXII diffractometer
Absorption correction: multi-scan (*SADABS*; Bruker, 2009 ▶) *T*_min_ = 0.592, *T*_max_ = 0.737
7679 measured reflections
2827 independent reflections
2713 reflections with *I* > 2σ(*I*)
*R*_int_ = 0.021

#### Refinement

*R*[*F* ^2^ > 2σ(*F* ^2^)] = 0.049
*wR*(*F* ^2^) = 0.152
*S* = 1.15
2827 reflections
227 parameters
H-atom parameters constrained
Δρ_max_ = 0.33 e Å^−3^
Δρ_min_ = −0.54 e Å^−3^

### 

Data collection: *APEX2* (Bruker, 2009 ▶); cell refinement: *SAINT* (Bruker, 2009 ▶); data reduction: *SAINT*; program(s) used to solve structure: *SHELXS97* (Sheldrick, 2008 ▶); program(s) used to refine structure: *SHELXL97* (Sheldrick, 2008 ▶); molecular graphics: *SHELXTL* (Sheldrick, 2008 ▶); software used to prepare material for publication: *SHELXTL*.

## Supplementary Material

Crystal structure: contains datablock(s) I, global. DOI: 10.1107/S1600536812022131/bh2435sup1.cif

Structure factors: contains datablock(s) I. DOI: 10.1107/S1600536812022131/bh2435Isup2.hkl

Supplementary material file. DOI: 10.1107/S1600536812022131/bh2435Isup3.cml

Additional supplementary materials:  crystallographic information; 3D view; checkCIF report

## Figures and Tables

**Table 1:** Hydrogen-bond geometry (Å, °)

*D*—H⋯*A*	*D*—H	H⋯*A*	*D*⋯*A*	*D*—H⋯*A*
O1′—H1′*B*⋯N2^i^	0.85	2.10	2.869 (3)	150
N1—H1*A*⋯O1′^ii^	0.86	1.91	2.765 (3)	170
O1′—H1′*A*⋯N3	0.85	2.36	3.077 (4)	143

Symmetry codes: (i) 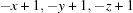; (ii) 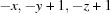.

**Fig. 1.**
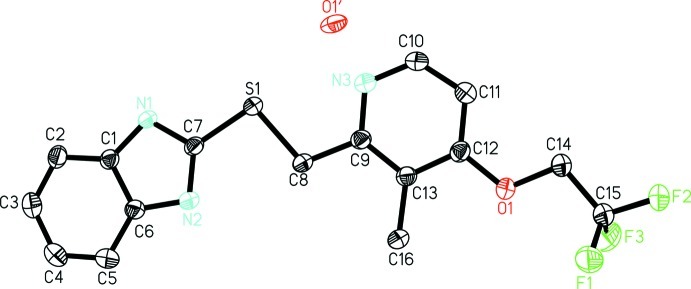
The content of the asymmetric unit of the title hydrate with displacement ellipsoids drawn at the 30% probability level. H atoms have been omitted for clarity.

**Fig. 2.**
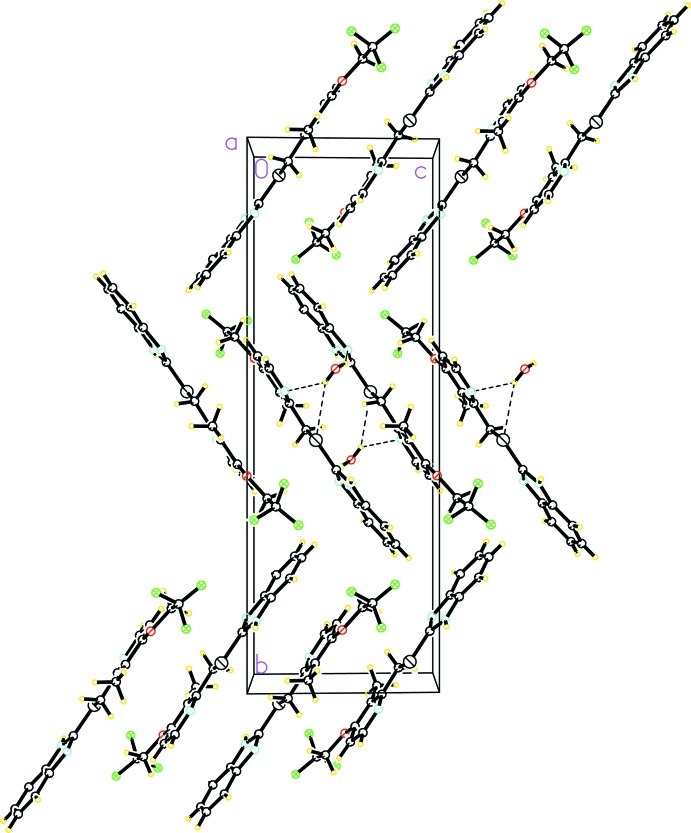
A packing diagram of the title compound. Supramolecular chains are running in the [100] direction, and dashed bonds indicate secondary hydrogen bonds connecting the chains in the crystal.

## supplementary crystallographic information

### Comment

The title compound is the critical reaction intermediate for the synthesis of lansoprazole (Del Rio *et al.*, 2007; Reddy *et al.*, 2008), and its analogs, used as drugs for their anti-ulcer effects (Iwahi *et al.*, 1991). Recently, the compound was successfully crystallized from 1-propanol, and the crystal structure is reported here. A first polymorph was X-ray characterized in space group *P*1 (Ren *et al.*, 2011).

The asymmetric unit (Fig. 1) contains one independent molecule and one water molecule which are involved in the formation of hydrogen-bonded chains *via*, N—H···O and O—H···N hydrogen bonds. These chains further interact through O—H···N(pyridine) contacts (Fig. 2). The water molecule could thus be considered to be a hydrogen-bond bridge, which provides stability to the crystal lattice. The hydrogen bond characteristics and geometric parameters are given in Table 1. The geometry of the main molecule is close to that reported for analog systems (Hakim Al-arique *et al.*, 2010; Swamy & Ravikumar, 2007).

### Experimental

The raw material was kindly provided by Shanghai Enran Sci-Tech Investment Management Co., Ltd. The compound was dissolved in 1-propanol and suitable crystals were obtained by slow evaporation at room temperature over a period of one week.

### Refinement

Water H atoms were initially located in a difference map and then fixed in their as-found positions, while all other H atoms were constrained to an ideal geometry with C—H distances of 0.93 Å (aromatic CH), 0.96 Å (methyl CH_3_), 0.97 Å (methylene CH_2_) and N—H distance of 0.86 Å (imidazolic NH). Isotropic displacement parameters for H atoms were calculated as *U*_iso_(H) = *xU*_eq_(carrier atom) with *x* = 1.5 (H_2_O and methyl group) or *x* = 1.2 (other H atoms).

### Crystal data

**Table:**

C_16_H_14_F_3_N_3_OS·H_2_O	*F*(000) = 768
*M**_r_* = 371.39	*D*_x_ = 1.481 Mg m^−^^3^
Monoclinic, *P*2_1_/*c*	Cu *K*α radiation, λ = 1.54178 Å
Hall symbol: -P 2ybc	Cell parameters from 2713 reflections
*a* = 7.3886 (15) Å	θ = 3.5–67.0°
*b* = 25.497 (5) Å	µ = 2.17 mm^−^^1^
*c* = 8.8579 (18) Å	*T* = 296 K
β = 93.64 (3)°	Column, colorless
*V* = 1665.4 (6) Å^3^	0.27 × 0.16 × 0.15 mm
*Z* = 4	

### Data collection

**Table:**

Bruker SMART APEXII diffractometer	2827 independent reflections
Radiation source: fine-focus sealed tube	2713 reflections with *I* > 2σ(*I*)
Graphite monochromator	*R*_int_ = 0.021
Detector resolution: 0 pixels mm^-1^	θ_max_ = 67.0°, θ_min_ = 3.5°
φ and ω scans	*h* = −8→8
Absorption correction: multi-scan (*SADABS*; Bruker, 2009)	*k* = −30→29
*T*_min_ = 0.592, *T*_max_ = 0.737	*l* = −9→10
7679 measured reflections	

### Refinement

**Table:**

Refinement on *F*^2^	Secondary atom site location: difference Fourier map
Least-squares matrix: full	Hydrogen site location: inferred from neighbouring sites
*R*[*F*^2^ > 2σ(*F*^2^)] = 0.049	H-atom parameters constrained
*wR*(*F*^2^) = 0.152	*w* = 1/[σ^2^(*F*_o_^2^) + (0.0686*P*)^2^ + 1.4537*P*] where *P* = (*F*_o_^2^ + 2*F*_c_^2^)/3
*S* = 1.15	(Δ/σ)_max_ = 0.001
2827 reflections	Δρ_max_ = 0.33 e Å^−^^3^
227 parameters	Δρ_min_ = −0.54 e Å^−^^3^
0 restraints	Extinction correction: *SHELXL97* (Sheldrick, 2008), Fc^*^=kFc[1+0.001xFc^2^λ^3^/sin(2θ)]^-1/4^
0 constraints	Extinction coefficient: 0.0160 (11)
Primary atom site location: structure-invariant direct methods	

### Fractional atomic coordinates and isotropic or equivalent isotropic displacement parameters (Å^2^)

**Table:**

	*x*	*y*	*z*	*U*_iso_*/*U*_eq_	
S1	0.30666 (8)	0.45480 (3)	0.64708 (8)	0.0437 (3)	
O1	0.8564 (3)	0.60957 (8)	0.9928 (2)	0.0480 (5)	
O1'	0.1360 (3)	0.58511 (9)	0.5422 (3)	0.0625 (6)	
H1'B	0.2211	0.5963	0.4904	0.094*	
H1'A	0.1850	0.5606	0.5956	0.094*	
N1	0.2125 (3)	0.37523 (8)	0.4607 (3)	0.0403 (5)	
H1A	0.0998	0.3838	0.4597	0.048*	
N2	0.5109 (3)	0.37741 (9)	0.5223 (3)	0.0450 (6)	
N3	0.3764 (3)	0.54450 (10)	0.8138 (3)	0.0485 (6)	
F1	1.1369 (3)	0.62171 (9)	1.1942 (3)	0.0881 (8)	
F2	1.0471 (3)	0.69647 (9)	1.2672 (3)	0.0814 (7)	
F3	1.1303 (3)	0.68563 (10)	1.0413 (3)	0.0862 (7)	
C1	0.2856 (3)	0.33433 (10)	0.3837 (3)	0.0394 (6)	
C2	0.2097 (4)	0.29744 (11)	0.2827 (4)	0.0494 (7)	
H2B	0.0858	0.2967	0.2566	0.059*	
C3	0.3268 (5)	0.26218 (12)	0.2231 (4)	0.0567 (8)	
H3B	0.2811	0.2374	0.1539	0.068*	
C4	0.5115 (5)	0.26269 (13)	0.2636 (4)	0.0628 (9)	
H4A	0.5861	0.2379	0.2221	0.075*	
C5	0.5867 (4)	0.29905 (13)	0.3639 (4)	0.0593 (8)	
H5A	0.7104	0.2991	0.3910	0.071*	
C6	0.4717 (4)	0.33567 (11)	0.4233 (3)	0.0442 (6)	
C7	0.3531 (3)	0.39957 (10)	0.5389 (3)	0.0389 (6)	
C8	0.5380 (3)	0.47338 (11)	0.7022 (3)	0.0437 (6)	
H8A	0.5965	0.4457	0.7622	0.052*	
H8B	0.6051	0.4784	0.6127	0.052*	
C9	0.5390 (3)	0.52327 (10)	0.7926 (3)	0.0394 (6)	
C10	0.3747 (4)	0.58788 (13)	0.8963 (4)	0.0550 (8)	
H10A	0.2631	0.6034	0.9101	0.066*	
C11	0.5275 (4)	0.61143 (12)	0.9630 (4)	0.0504 (7)	
H11A	0.5192	0.6411	1.0233	0.060*	
C12	0.6938 (4)	0.58936 (10)	0.9371 (3)	0.0411 (6)	
C13	0.7032 (3)	0.54422 (10)	0.8491 (3)	0.0391 (6)	
C14	0.8503 (4)	0.65056 (11)	1.1002 (4)	0.0471 (7)	
H14A	0.7841	0.6394	1.1857	0.056*	
H14B	0.7905	0.6811	1.0549	0.056*	
C15	1.0414 (4)	0.66317 (12)	1.1500 (4)	0.0550 (8)	
C16	0.8805 (4)	0.51935 (12)	0.8153 (4)	0.0503 (7)	
H16A	0.9786	0.5392	0.8632	0.075*	
H16B	0.8918	0.5189	0.7079	0.075*	
H16C	0.8845	0.4841	0.8532	0.075*	

### Atomic displacement parameters (Å^2^)

**Table:**

	*U*^11^	*U*^22^	*U*^33^	*U*^12^	*U*^13^	*U*^23^
S1	0.0305 (4)	0.0399 (4)	0.0604 (5)	−0.0018 (2)	0.0018 (3)	−0.0058 (3)
O1	0.0399 (10)	0.0434 (11)	0.0605 (13)	−0.0046 (8)	0.0004 (9)	−0.0123 (9)
O1'	0.0317 (10)	0.0713 (15)	0.0844 (16)	0.0044 (10)	0.0033 (10)	0.0137 (12)
N1	0.0268 (10)	0.0389 (12)	0.0549 (14)	−0.0020 (9)	−0.0001 (9)	0.0003 (10)
N2	0.0311 (11)	0.0432 (13)	0.0604 (15)	0.0009 (9)	0.0002 (10)	−0.0017 (11)
N3	0.0340 (12)	0.0533 (15)	0.0583 (15)	0.0010 (10)	0.0034 (11)	−0.0086 (11)
F1	0.0793 (15)	0.0701 (14)	0.1096 (18)	0.0313 (11)	−0.0360 (13)	−0.0204 (12)
F2	0.0623 (12)	0.0790 (14)	0.1000 (17)	0.0072 (10)	−0.0181 (11)	−0.0482 (13)
F3	0.0635 (13)	0.0855 (16)	0.1106 (18)	−0.0273 (12)	0.0131 (12)	−0.0078 (14)
C1	0.0371 (14)	0.0354 (13)	0.0455 (14)	−0.0030 (10)	0.0013 (11)	0.0058 (11)
C2	0.0482 (16)	0.0424 (15)	0.0570 (18)	−0.0087 (12)	−0.0022 (13)	0.0007 (13)
C3	0.069 (2)	0.0429 (16)	0.0582 (19)	−0.0049 (15)	0.0002 (15)	−0.0058 (14)
C4	0.067 (2)	0.0494 (18)	0.073 (2)	0.0103 (16)	0.0083 (17)	−0.0107 (16)
C5	0.0447 (17)	0.0555 (19)	0.078 (2)	0.0098 (14)	0.0025 (15)	−0.0061 (16)
C6	0.0374 (14)	0.0377 (14)	0.0577 (17)	0.0010 (11)	0.0036 (12)	0.0029 (12)
C7	0.0302 (12)	0.0360 (13)	0.0504 (15)	−0.0030 (10)	0.0008 (11)	0.0059 (11)
C8	0.0298 (13)	0.0415 (14)	0.0595 (17)	−0.0010 (11)	−0.0004 (11)	−0.0026 (12)
C9	0.0342 (13)	0.0369 (13)	0.0471 (15)	−0.0007 (10)	0.0034 (11)	0.0036 (11)
C10	0.0366 (15)	0.0595 (19)	0.069 (2)	0.0064 (13)	0.0065 (14)	−0.0149 (15)
C11	0.0450 (16)	0.0470 (16)	0.0594 (18)	0.0022 (13)	0.0052 (13)	−0.0100 (13)
C12	0.0380 (14)	0.0381 (14)	0.0468 (15)	−0.0045 (11)	0.0007 (11)	0.0026 (11)
C13	0.0347 (14)	0.0347 (14)	0.0481 (16)	−0.0005 (10)	0.0029 (11)	0.0046 (10)
C14	0.0461 (16)	0.0379 (14)	0.0569 (17)	−0.0021 (12)	0.0008 (13)	−0.0065 (12)
C15	0.0499 (17)	0.0446 (16)	0.069 (2)	0.0029 (13)	−0.0052 (15)	−0.0146 (14)
C16	0.0331 (14)	0.0481 (16)	0.069 (2)	−0.0004 (12)	−0.0021 (13)	−0.0076 (14)

### Geometric parameters (Å, º)

**Table:**

S1—C7	1.750 (3)	C3—H3B	0.9300
S1—C8	1.811 (3)	C4—C5	1.376 (5)
O1—C12	1.370 (3)	C4—H4A	0.9300
O1—C14	1.416 (3)	C5—C6	1.388 (4)
O1'—H1'B	0.8499	C5—H5A	0.9300
O1'—H1'A	0.8497	C8—C9	1.503 (4)
N1—C7	1.361 (3)	C8—H8A	0.9700
N1—C1	1.375 (4)	C8—H8B	0.9700
N1—H1A	0.8600	C9—C13	1.390 (4)
N2—C7	1.312 (3)	C10—C11	1.378 (4)
N2—C6	1.397 (4)	C10—H10A	0.9300
N3—C10	1.326 (4)	C11—C12	1.383 (4)
N3—C9	1.342 (4)	C11—H11A	0.9300
F1—C15	1.317 (4)	C12—C13	1.394 (4)
F2—C15	1.340 (4)	C13—C16	1.502 (4)
F3—C15	1.329 (4)	C14—C15	1.488 (4)
C1—C2	1.392 (4)	C14—H14A	0.9700
C1—C6	1.398 (4)	C14—H14B	0.9700
C2—C3	1.376 (5)	C16—H16A	0.9600
C2—H2B	0.9300	C16—H16B	0.9600
C3—C4	1.389 (5)	C16—H16C	0.9600
			
C7—S1—C8	98.25 (13)	H8A—C8—H8B	108.2
C12—O1—C14	117.0 (2)	N3—C9—C13	124.3 (3)
H1'B—O1'—H1'A	104.1	N3—C9—C8	116.2 (2)
C7—N1—C1	106.6 (2)	C13—C9—C8	119.5 (2)
C7—N1—H1A	126.7	N3—C10—C11	124.3 (3)
C1—N1—H1A	126.7	N3—C10—H10A	117.9
C7—N2—C6	104.4 (2)	C11—C10—H10A	117.9
C10—N3—C9	117.0 (3)	C10—C11—C12	117.6 (3)
N1—C1—C2	132.6 (3)	C10—C11—H11A	121.2
N1—C1—C6	105.6 (2)	C12—C11—H11A	121.2
C2—C1—C6	121.9 (3)	O1—C12—C11	123.7 (3)
C3—C2—C1	116.8 (3)	O1—C12—C13	116.0 (2)
C3—C2—H2B	121.6	C11—C12—C13	120.3 (3)
C1—C2—H2B	121.6	C9—C13—C12	116.4 (2)
C2—C3—C4	121.8 (3)	C9—C13—C16	121.2 (2)
C2—C3—H3B	119.1	C12—C13—C16	122.3 (2)
C4—C3—H3B	119.1	O1—C14—C15	106.8 (2)
C5—C4—C3	121.5 (3)	O1—C14—H14A	110.4
C5—C4—H4A	119.2	C15—C14—H14A	110.4
C3—C4—H4A	119.2	O1—C14—H14B	110.4
C4—C5—C6	117.8 (3)	C15—C14—H14B	110.4
C4—C5—H5A	121.1	H14A—C14—H14B	108.6
C6—C5—H5A	121.1	F1—C15—F3	106.3 (3)
C5—C6—N2	130.0 (3)	F1—C15—F2	106.8 (3)
C5—C6—C1	120.3 (3)	F3—C15—F2	107.2 (3)
N2—C6—C1	109.7 (2)	F1—C15—C14	113.3 (3)
N2—C7—N1	113.7 (2)	F3—C15—C14	112.4 (3)
N2—C7—S1	127.9 (2)	F2—C15—C14	110.5 (3)
N1—C7—S1	118.38 (19)	C13—C16—H16A	109.5
C9—C8—S1	109.75 (18)	C13—C16—H16B	109.5
C9—C8—H8A	109.7	H16A—C16—H16B	109.5
S1—C8—H8A	109.7	C13—C16—H16C	109.5
C9—C8—H8B	109.7	H16A—C16—H16C	109.5
S1—C8—H8B	109.7	H16B—C16—H16C	109.5
			
C7—N1—C1—C2	−177.4 (3)	C10—N3—C9—C13	−1.1 (4)
C7—N1—C1—C6	1.2 (3)	C10—N3—C9—C8	178.5 (3)
N1—C1—C2—C3	178.3 (3)	S1—C8—C9—N3	−0.8 (3)
C6—C1—C2—C3	0.0 (4)	S1—C8—C9—C13	178.8 (2)
C1—C2—C3—C4	1.1 (5)	C9—N3—C10—C11	−1.3 (5)
C2—C3—C4—C5	−0.9 (6)	N3—C10—C11—C12	2.6 (5)
C3—C4—C5—C6	−0.3 (5)	C14—O1—C12—C11	9.6 (4)
C4—C5—C6—N2	−177.8 (3)	C14—O1—C12—C13	−170.9 (2)
C4—C5—C6—C1	1.3 (5)	C10—C11—C12—O1	178.1 (3)
C7—N2—C6—C5	179.0 (3)	C10—C11—C12—C13	−1.4 (4)
C7—N2—C6—C1	−0.3 (3)	N3—C9—C13—C12	2.1 (4)
N1—C1—C6—C5	−179.9 (3)	C8—C9—C13—C12	−177.5 (2)
C2—C1—C6—C5	−1.2 (4)	N3—C9—C13—C16	−177.5 (3)
N1—C1—C6—N2	−0.6 (3)	C8—C9—C13—C16	3.0 (4)
C2—C1—C6—N2	178.1 (3)	O1—C12—C13—C9	179.7 (2)
C6—N2—C7—N1	1.1 (3)	C11—C12—C13—C9	−0.7 (4)
C6—N2—C7—S1	−179.2 (2)	O1—C12—C13—C16	−0.7 (4)
C1—N1—C7—N2	−1.5 (3)	C11—C12—C13—C16	178.8 (3)
C1—N1—C7—S1	178.72 (18)	C12—O1—C14—C15	176.4 (2)
C8—S1—C7—N2	6.6 (3)	O1—C14—C15—F1	−51.6 (4)
C8—S1—C7—N1	−173.7 (2)	O1—C14—C15—F3	69.0 (3)
C7—S1—C8—C9	176.9 (2)	O1—C14—C15—F2	−171.3 (3)

### Hydrogen-bond geometry (Å, º)

**Table:**

*D*—H···*A*	*D*—H	H···*A*	*D*···*A*	*D*—H···*A*
O1′—H1′*B*···N2^i^	0.85	2.10	2.869 (3)	150
N1—H1*A*···O1′^ii^	0.86	1.91	2.765 (3)	170
O1′—H1′*A*···N3	0.85	2.36	3.077 (4)	143
O1′—H1′*A*···S1	0.85	2.87	3.653 (2)	154

Symmetry codes: (i) −*x*+1, −*y*+1, −*z*+1; (ii) −*x*, −*y*+1, −*z*+1.
